# Stimulation enhancement effect of the combination of exoskeleton-assisted hand rehabilitation and fingertip haptic stimulation

**DOI:** 10.3389/fnins.2023.1149265

**Published:** 2023-05-23

**Authors:** Min Li, Jing Chen, Bo He, Guoying He, Chen-Guang Zhao, Hua Yuan, Jun Xie, Guanghua Xu, Jichun Li

**Affiliations:** ^1^School of Mechanical Engineering, Xi’an Jiaotong University, Xi’an, Shaanxi, China; ^2^Department of Rehabilitation, Xijing Hospital, Fourth Military Medical University, Xi’an, China; ^3^School of Computing, Newcastle University, Newcastle upon Tyne, United Kingdom

**Keywords:** haptic feedback, hand rehabilitation, fingertip haptic stimulation, robot-assisted hand rehabilitation, event-related potential (ERP)

## Abstract

**Introduction:**

Providing stimulation enhancements to existing hand rehabilitation training methods may help stroke survivors achieve better treatment outcomes. This paper presents a comparison study to explore the stimulation enhancement effects of the combination of exoskeleton-assisted hand rehabilitation and fingertip haptic stimulation by analyzing behavioral data and event-related potentials.

**Methods:**

The stimulation effects of the touch sensations created by a water bottle and that created by cutaneous fingertip stimulation with pneumatic actuators are also investigated. Fingertip haptic stimulation was combined with exoskeleton-assisted hand rehabilitation while the haptic stimulation was synchronized with the motion of our hand exoskeleton. In the experiments, three experimental modes, including exoskeleton-assisted grasping motion without haptic stimulation (Mode 1), exoskeleton-assisted grasping motion with haptic stimulation (Mode 2), and exoskeleton-assisted grasping motion with a water bottle (Mode 3), were compared.

**Results:**

The behavioral analysis results showed that the change of experimental modes had no significant effect on the recognition accuracy of stimulation levels (*p* = 0.658), while regarding the response time, exoskeleton-assisted grasping motion with haptic stimulation was the same as grasping a water bottle (*p* = 0.441) but significantly different from that without haptic stimulation (*p* = 0.006). The analysis of event-related potentials showed that the primary motor cortex, premotor cortex, and primary somatosensory areas of the brain were more activated when both the hand motion assistance and fingertip haptic feedback were provided using our proposed method (P300 amplitude 9.46 μV). Compared to only applying exoskeleton-assisted hand motion, the P300 amplitude was significantly improved by providing both exoskeleton-assisted hand motion and fingertip haptic stimulation (*p* = 0.006), but no significant differences were found between any other two modes (Mode 2 vs. Mode 3: *p* = 0.227, Mode 1 vs. Mode 3: *p* = 0.918). Different modes did not significantly affect the P300 latency (*p* = 0.102). Stimulation intensity had no effect on the P300 amplitude (*p* = 0.295, 0.414, 0.867) and latency (*p* = 0.417, 0.197, 0.607).

**Discussion:**

Thus, we conclude that combining exoskeleton-assisted hand motion and fingertip haptic stimulation provided stronger stimulation on the motor cortex and somatosensory cortex of the brain simultaneously; the stimulation effects of the touch sensations created by a water bottle and that created by cutaneous fingertip stimulation with pneumatic actuators are similar.

## 1. Introduction

Stroke is a severe brain disorder and one of the leading causes of acquired disability (Murphy and Werring, 2020). Impaired hand function is one of the most common effects of stroke. Many stroke survivors suffer from hand motor dysfunctions, and thus their abilities to live independently are greatly affected (Colombo et al., 2019). Hand rehabilitation training is an important means to help patients regain their hand functions (Baniqued et al., 2021). Robot-assisted rehabilitation can provide high-dose, high-intensity interventions, quantitatively monitor patient performance, adjust rehabilitation training according to patients’ progress, and ensure consistency in planning a therapy program. Those are significant advantages of robotic-assisted devices over conventional training methods relied on therapists (Shi et al., 2019; Hobbs and Artemiadis, 2020; Morone et al., 2020). In recent years, as a type of hand rehabilitation robot, hand exoskeleton has attracted extensive research attention. The commonly used exoskeleton-assisted rehabilitation training method, continuous passive motion (CPM) training (Almusawi and Husi, 2021; Nasrallah et al., 2021), involving repetitive tasks such as grasping a water bottle, can provide sensorimotor feedback during the process and has been shown to be effective in hand motor function improvements (Li et al., 2021).

The essence of rehabilitation of hand motor dysfunction after a stroke is the reconstruction of motor control and feedback loop (Ethier et al., 2015; Li et al., 2018). It has been proved that active enrollment in rehabilitation training can lead to better treatment outcomes (Kai Keng and Cuntai, 2013; Teo and Chew, 2014). However, exoskeleton-assisted CPM grasping training is passive, and thus it is difficult for the patient to stay focused during the training process. Moreover, stroke patients with hand dysfunction may also lose part of somatosensory functions, including haptic sensation and proprioceptive sensation (Kessner et al., 2019). Somatosensory dysfunction can greatly affect the rehabilitation effect of patients (Findlater et al., 2019). Nevertheless, exoskeleton-assisted CPM grasping training can only provide limited sensorimotor stimulation to the patient. Therefore, to achieve better treatment outcomes, it is necessary to seek for means to improve patients’ active participation and to provide rich sensory stimulations in exoskeleton-assisted CPM grasping training.

Multimodal sensory feedback, which refers to the use of multiple sensory modalities such as visual, auditory, and haptic feedback to provide a more comprehensive and effective feedback experience, during rehabilitation training can enrich the patient’s experience to improve training involvement, enhance motor learning, help rebuild the sensorimotor loop, and thus promote functional recovery of patients’ limbs (Norwood et al., 2018; Sharififar et al., 2018; Winkler et al., 2022). Virtual reality (VR)-mediated rehabilitation is a useful tool to achieve multimodal sensory feedback and attract active involvement of patients (Feng et al., 2019). In VR-mediated rehabilitation, the patient is often required to complete tasks of grasping a virtual object. When the patient interacts with the virtual object, corresponding haptic feedback is added to the patient’s hand creating the touch sensation. Cutaneous haptic (also can be referred as tactile) inputs, which are generated by stimulating mechanoreceptors in the skin, and detect skin contact with objects and perception of surface properties (Lim et al., 2015), can be used in such rehabilitation training.

Combining cutaneous haptic stimulation to the fingertips with exoskeleton-assisted hand rehabilitation may provide sensorimotor and cutaneous haptic feedback simultaneously. It may have the potential to improve the training involvement of stroke patients and thus promote the restoration of motor function. In our previous study, we presented the creation and validation of a fingertip cutaneous haptic stimulation system for exoskeleton-assisted hand rehabilitation using 3D-printed pneumatic actuators to improve the training involvement of stroke patients and promote motor function recovery (Li et al., 2021). For the first time, fingertip cutaneous haptic stimulation was integrated with the hand exoskeleton to form a hand rehabilitation system. During a CPM glass-grasping training process assisted by a hand exoskeleton, fingertip haptic stimulation was added when the hand touched a simulated virtual glass imitating the contact force of grasping a glass of water. Our experimental results proved that adding haptic stimulation to exoskeleton-assisted hand movements significantly increased the attention levels of the participants (Li et al., 2021). Further investigation is needed on the effects of combining exoskeleton-assisted hand motion and fingertip haptic stimulation on the motor cortex and somatosensory cortex of the brain. It is also necessary to objectively evaluate the brain stimulation effects of the touch sensations created by interacting with real objects and that created by adding haptic stimulation when interacting with virtual objects. To the best of our knowledge, such research has not been reported.

Event-related potentials (ERPs) in electroencephalographic (EEG) signals, consisting of exogenous and endogenous components, represent the brain activities both internally and externally (Olichney et al., 2022). While the exogenous components of ERPs show the effect of involuntary attention related to the physical characteristics of the external stimulus, the endogenous component reveals the psychological reaction to the stimulus (Donoghue and Voytek, 2022). There are studies using ERPs to investigate the somatosensory response to cutaneous haptic stimulations (Zhang et al., 2016; Chen and Ge, 2017; Tang et al., 2020). P300, appears in the transition stage between exogenous and endogenous components, is related to exogenous stimuli. At the same time, P300 is highly related to the brain cognitive process which is mainly affected by attention resource allocation, memory updating, and inhibitory processing in the brain. Therefore, P300 provides an effective way for studying the mechanism of brain nervous system activity associated with the human cognitive process in response to stimuli (Salehinejad et al., 2021; Tao et al., 2022). The research on P300 mainly studies the amplitude and latency of the wave crest. The main latency period of P300 occurs between 250 and 600 ms (Olichney et al., 2022). It is related to task processing demands, the concentration level and the cognitive capability of the subject (Tao et al., 2022). The latency of P300 reflects the speed of task completion, and shorter P300 latency is considered to have better cognitive performance (Chi et al., 2019; Olichney et al., 2022). The amplitude of P300 reflects the intensity of a person’s response to a stimulus, and the larger the amplitude, the stronger the response (Linden, 2005; Polich, 2007; Franken et al., 2011). Therefore, the amplitude and latency of P300 can be used as indicators to quantify the somatosensory stimulation effect of exoskeleton-assisted hand rehabilitation and fingertip haptic stimulation.

In this work, a comparison study is conducted to explore the stimulation enhancement effects of the combination of exoskeleton-assisted hand rehabilitation and fingertip haptic stimulation using both behavioral and ERP analyses. To the best of our knowledge, no similar studies have been reported. Moreover, for the first time, the stimulation effects of the touch sensations created by a water bottle and that created by cutaneous fingertip stimulation with pneumatic actuators are also compared using both behavioral and ERP analyses. For the behavioral analysis, the stimulation level recognition accuracy and response time are analyzed. For the ERP analysis, the amplitude and latency of P300 are examined.

The arrangement of the remainder of this paper is as follows. Section 2.1 describes the system design. Section 2.2 provides the experimental protocol. The experimental results are analyzed in section 3. Discussions are provided in Section 4.

## 2. Materials and methods

### 2.1. Hand rehabilitation system

**Figure 1:**
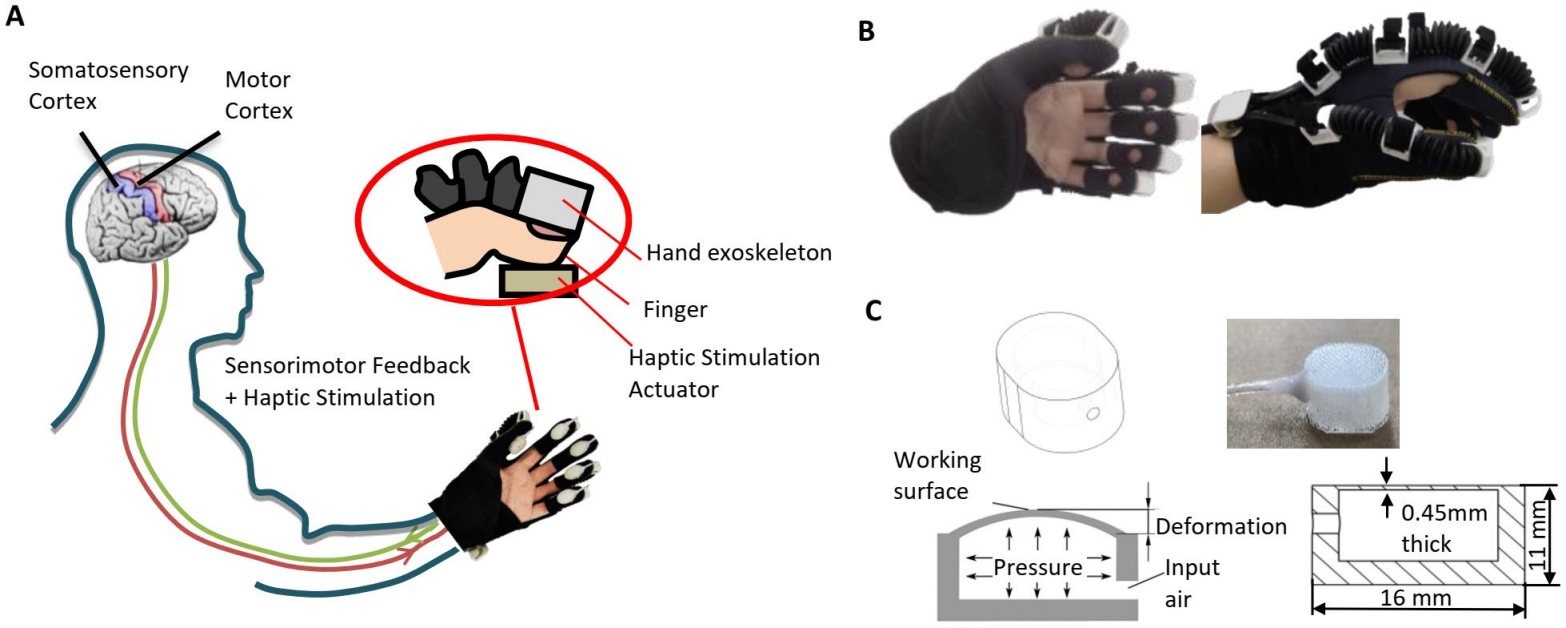
Illustrations of **(A)** our hand rehabilitation robot system combining hand exoskeleton and fingertip haptic stimulation, **(B)** hand exoskeleton, and **(C)** pneumatic haptic stimulation actuator.

**Figure 2:**
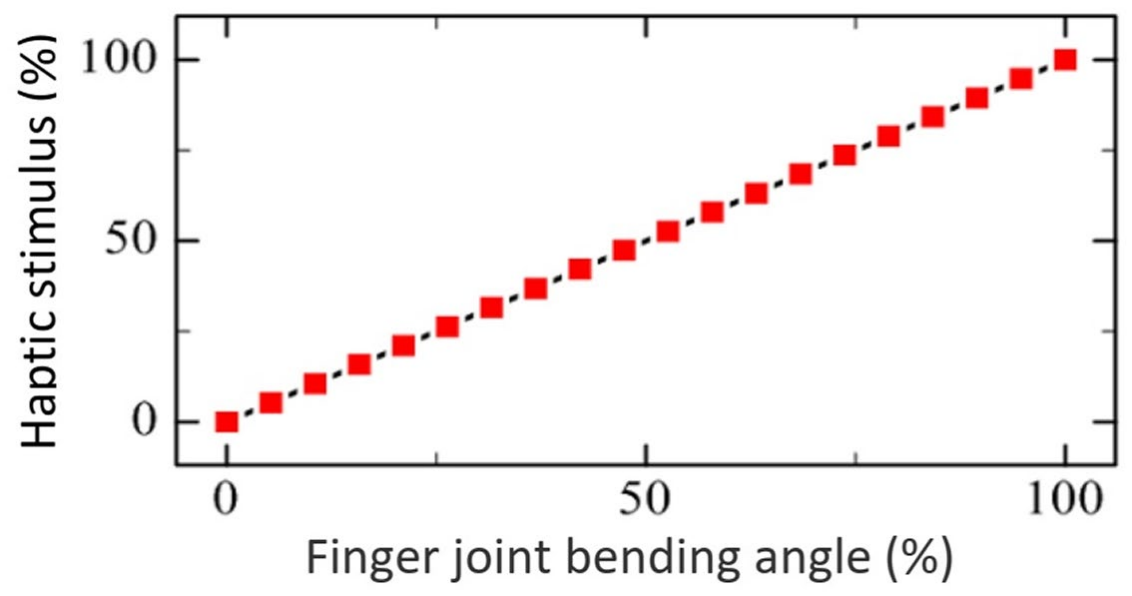
The relationship between the fingertip haptic stimulation force and the grasping process.

As shown in Figure 1A, the combination of exoskeleton-assisted hand rehabilitation and fingertip haptic stimulation is designed to improve the patient’s involvement in the training process, and thus (i) to enhance motor learning, (ii) to help the recovery of a sensorimotor feedback loop, and (iii) to promote the recovery of hand motor function. A pneumatic hand exoskeleton is controlled to drag the user’s hand conducting a grasping motion resulting in motor sensory feedback. During the process, haptic stimulation actuators mounted on the fingertips create contact forces between the actuators and the fingertips enhancing patient’s somatosensory stimulation. The generated fingertip force is set to be proportional to the grasping process (see Figure 2).

The pneumatic hand exoskeleton (see Figure 1B) contains five air bellows (with the outside large and small diameters of 15 and 9 mm, respectively) attached at the back of the fingers. The air bellows are fabricated using injection molding with a material of TPU. All five fingers are controlled by one air channel to provide grasping motion assistance. The weight of the hand exoskeleton is 120 g. The relationship between the input air pressure to the hand exoskeleton and the average bending angle of the MCP joints of the five fingers in the grasping motion can be expressed as


(1) $$A=\frac{3}{5}P_{1}$$

where *P_1_* is the pressure in the air chamber of the hand exoskeleton with the unit of kPa and *A* is the average bending angle of the MCP joints of the five fingers in the grasping motion. The maximum air pressure of the hand exoskeleton is 100 kPa and the corresponding average MCP joint angle of the five fingers is 60°.

Haptic simulation actuators are mounted on the fingertips. As shown in Figure 1C, each haptic stimulation actuator contains an air chamber. When air is injected into the air chamber, the working surface inflates to generate force to the fingertip. The detailed design and fabrication process were reported in our previous research (Li et al., 2021). The actuators used here are smaller than those used in our previous research (Li et al., 2021; length: 16 vs. 20 mm, height: 11 vs. 16 mm). This actuator can create a contact force to the fingertip up to 12 N. The relationship between the input air pressure can the output force can be expressed as


(2) $$F=0.03767P_{2}-0.1694$$

where *P_2_* is the pressure in the air chamber of the haptic simulation actuator with the unit of kPa and *F* is the created contact force.

**Figure 3:**
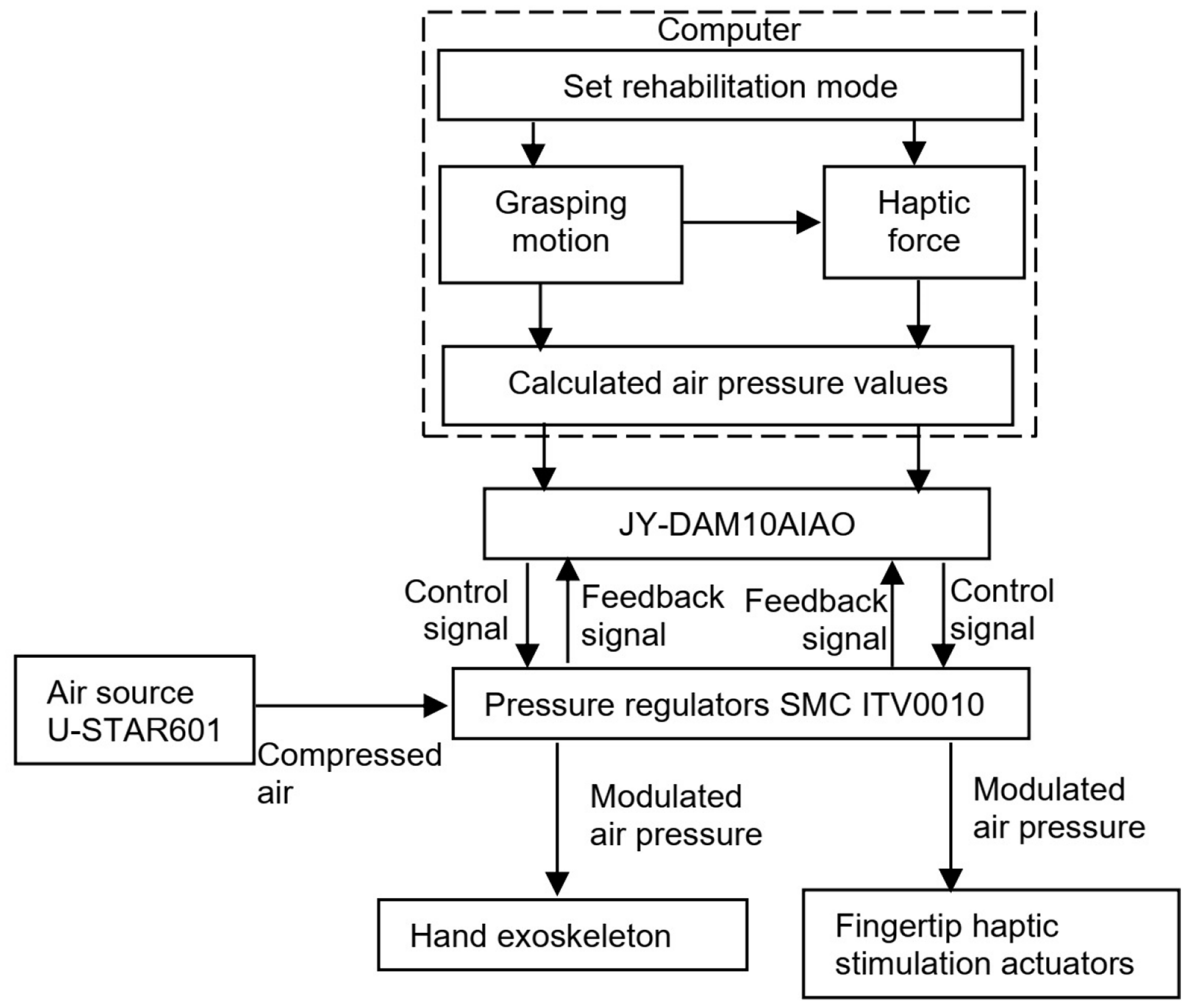
System integration and control of the hand rehabilitation combining the hand exoskeleton and the fingertip haptic stimulation.

Figure 3 shows the system integration and control of the hand rehabilitation system. Pressurized air is provided by an air compressor (U-STAR601, U-STAR, China). When the computer sends a start command, the analog input/output module JY-DAM10AIAO (Beijing Elit Gathering Electron, Beijing, China) starts to send the control signals to the pressure regulators to control the hand exoskeleton. The target haptic force is calculated according to the relationship between the haptic force and the grasping motion process defined in Figure 2, and the transfers to the analog input/output module JY-DAM10AIAO to control the air pressure inside the fingertip haptic stimulation actuators via the pressure regulators SMC ITV0010. Pressurized air is provided by an air compressor U-STAR601. The feedback signals from the pressure regulators are monitored by the JY-DAM10AIAO device.

### 2.2. Experimental protocol

**Figure 4:**
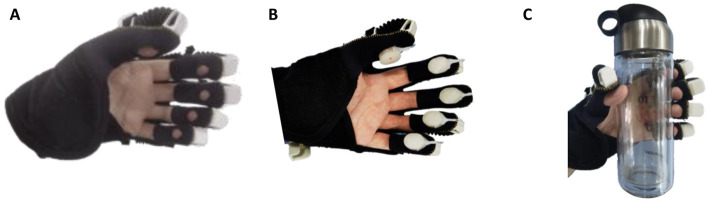
Three experimental modes: **(A)** exoskeleton-assisted grasping motion without haptic stimulation, **(B)** exoskeleton-assisted grasping motion with haptic stimulation, and **(C)** exoskeleton-assisted grasping with a water bottle.

To study the influence of cutaneous haptic feedback combined with motor feedback on relevant brain regions and the difference of the influence brought by different fingertip cutaneous haptic stimulations, this paper took fingertip cutaneous haptic stimulation as the only variable, designed a controlled experiment, and extracted the behavioral data and EPRs of the participants in three experimental modes for analysis. As shown in Figure 4, those three experimental modes include (1) exoskeleton-assisted grasping motion without haptic stimulation, (2) exoskeleton-assisted grasping motion with haptic stimulation, and (3) exoskeleton-assisted grasping motion with a water bottle. The fingertip haptic stimulation starts with the activation of the hand exoskeleton in mode 2 while the fingertip haptic stimulation starts until the fingertips touch the water bottle in mode 3. In VR-mediated rehabilitation, the patient is often required to complete tasks of grasping a virtual object. When they interact with the virtual object, corresponding haptic feedback is added to the user’s hand creating the touch sensation mimicking grasping a real object. Grasping a water bottle is very common in our daily life and grasping a column shaped object is commonly used task in VR-mediated rehabilitation. Therefore, we designed mode 3 and used a water bottle in mode 3 to investigate the stimulation effects of the touch sensations created by a water bottle and that created by cutaneous fingertip stimulation.

**Figure 5:**
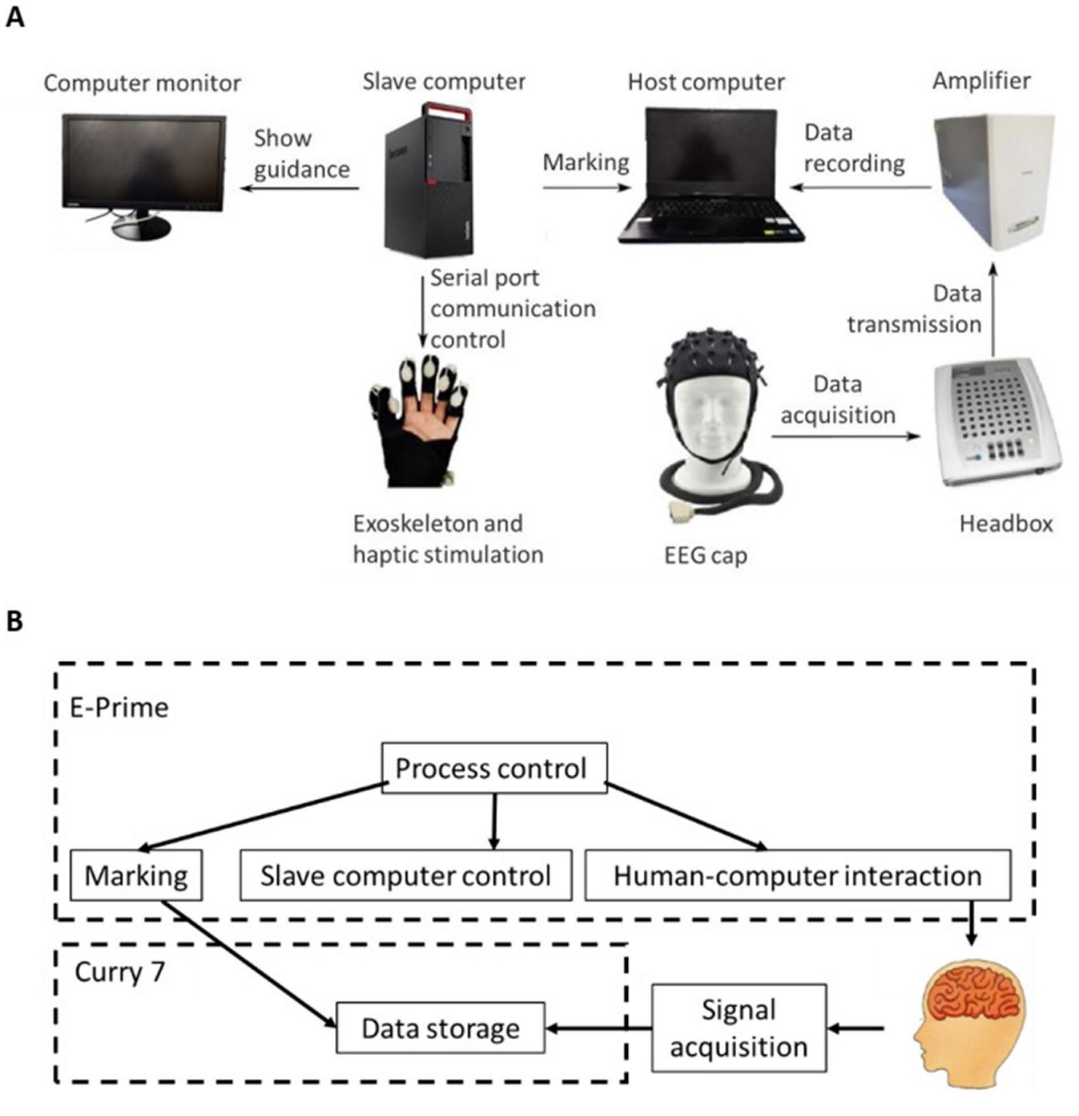
The experimental platform: **(A)** hardware and **(B)** software architecture.

The hardware of the experimental platform is shown in Figure 5A. During the experiment, the participants’ EEG data were monitored in real time at a frequency of 1 kHz by using a Neuroscan Quik-Cap EEG detection device (Compumedics Limited, Victoria, Australia) that can acquire 64-channel EEG data. As shown in Figure 5B, the software of the experimental platform was developed mainly based on CURRY 7 (Compumedics Limited, Victoria, Australia) and E-Prime (Psychology Software Tools, Sharpsburg, United States). Since ERPs generally occur a few hundred milliseconds after the stimulation, it is necessary to obtain the accurate time when the stimulus occurs. When the pneumatic haptic actuator or pneumatic exoskeleton needs to be activated during the experimental process, the software first sends control commands to the DAM module and then marks the beginning of the stimulation. At the end of the simulation, the end control command is sent again to the pneumatic haptic actuator or pneumatic exoskeleton, and then the end of the stimulation is marked. The behavioral data such as keystroke information and response time are recorded.

**Figure 6:**
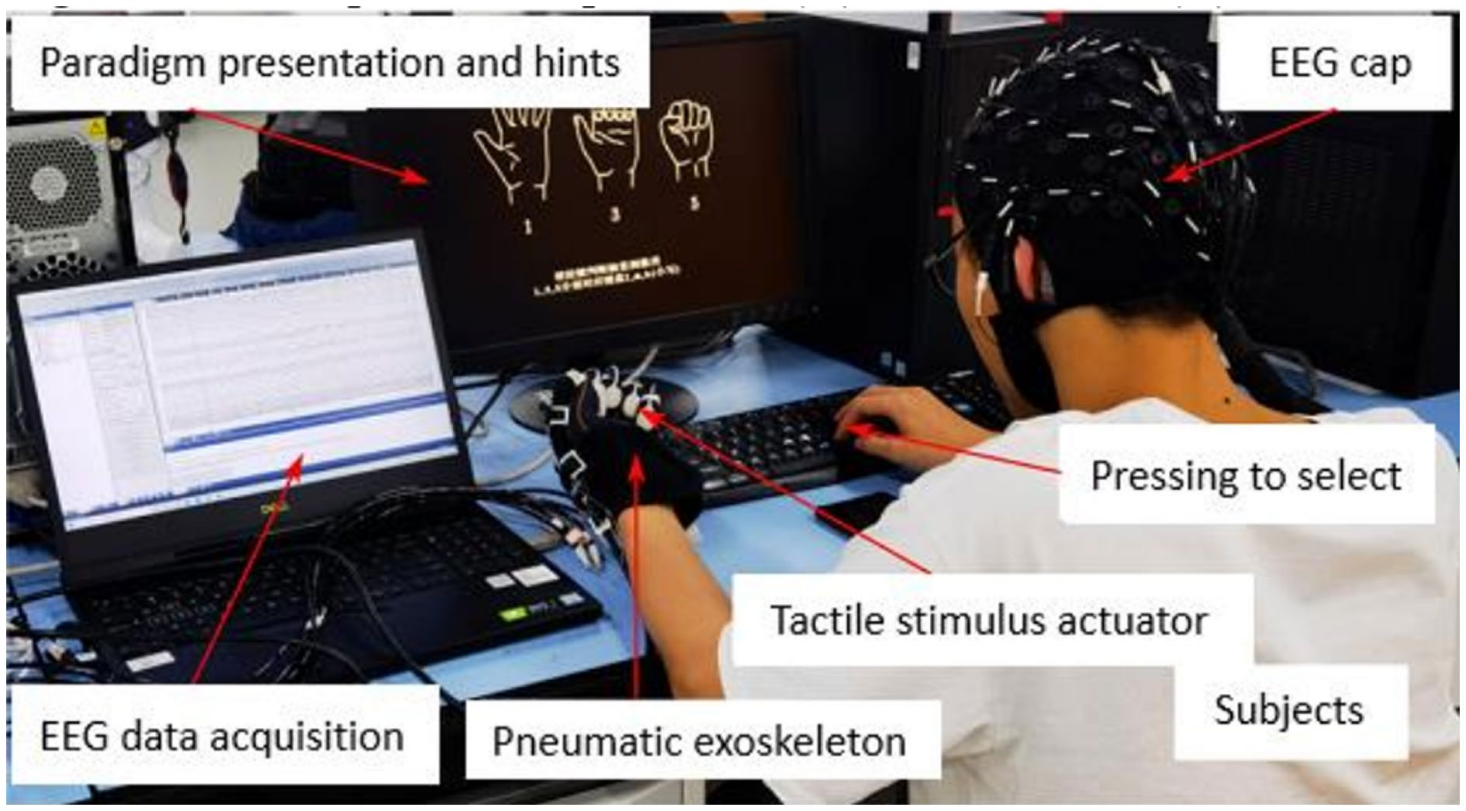
Experimental set-up.

**Figure 7:**
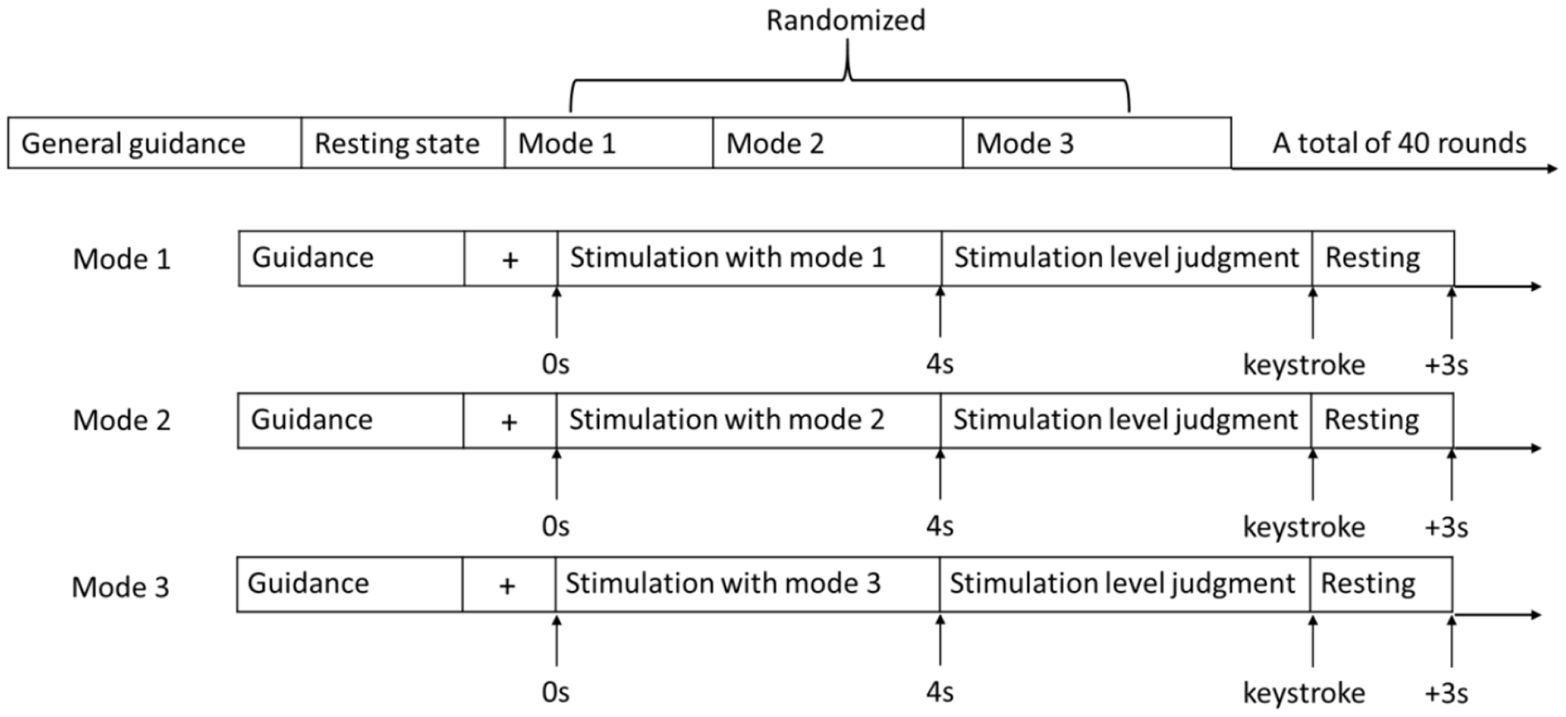
Experimental procedure.

The experimental setup is shown in Figure 6. Three different levels of stimulation were used (see Table 1). As can be seen from Table 1, the generated average fingertip contact forces of each stimulation level in Mode 3 was slightly higher than that in Mode 2. But the difference was not much. Therefore, we consider that the average fingertip contact forces of Mode 2 and Mode 3 are similar for each stimulation level. In our previous study, an experiment was conducted to investigate the fingertip contact forces during the process of grasping a glass and we found that the average peak forces when grasping a 150, 200, 250, and 300 g glass for the five fingers were within the range between 0.59 N and 3.43 N (Li et al., 2021). In this study, the average fingertip contact forces in Mode 2 and 3 were similar to this range. Eight healthy participants (all right-handed males with an average age of 24.5 and without prior experience with the system) were involved in this user study. Before the experiment, there was a process for the participants to get familiar with the different levels of stimulation. In this process, different levels in three experimental modes were showed to the participant. This process was repeated five times. During the experiment, all participants wore the pneumatic exoskeleton on their left hands and used their right hands for other operations. As shown in Figure 7, after recording the participant’s information, the experiment began and the general guidance of the experiment appeared on the computer monitor. When the participant pressed any key, the system started collecting resting EEG data. During this collection process, the participants were required to relax and remain as still as possible. Once the data collection was complete, the participants pressed any button to proceed to the formal experimental stage. After the formal experiment began, each stimulus was preceded by a prompt guidance displayed on the computer monitor. At this time, the participants were required to keep still and not blink until the prompt disappeared and the button selection interface appeared. The participants were asked to identify the stimulation level by pressing the corresponding ‘l’, ‘m’ and ‘h’ keys on the keyboard representing the stimulation levels from low to high. The maximum lasting time of the stimulation was 4 s. When the participant pressed any of those keys or the maximum stimulation time was reached, the fingertip haptic stimulation disappeared and the exoskeleton was reset. Then the participants had a three-second resting time before the next round of stimulation. Each round of the experiment contained one trial of three levels of stimulation. Those three different levels of stimulation were provided randomly. The experiment was repeated 40 times. At the end of every five rounds of the experiment, the participants were given a longer break up to 30 min.

**Table 1:** Three different levels of stimulation.

Stimulation levels	Air pressure in hand exoskeleton (kPa)	Average bending angles of the MCP joint (°)	Air pressure in haptic actuators (kPa)	Average fingertip contact force in Mode 2 (*N*)	Average fingertip contact force in Mode 3 (*N*)
Low	20	12	20	0.84	1.48
Medium	60	36	60	2.40	2.61
High	100	60	100	3.67	3.84

### 2.3. Experimental data analysis methods

Both behavioral and ERP analyses were performed to investigate the stimulation enhancement effects of the combination of exoskeleton-assisted hand rehabilitation and fingertip haptic stimulation. Behavioral analysis was conducted on the stimulation level recognition accuracy and response time. Recognition accuracy was calculated as the ratio of the number of the stimulation levels correctly identified by the participant to the total number of stimulations. The response time was defined as the time from the appearance of the selection interface on the computer monitor to the keystroke action. The data points when the stimulation level was misidentified were removed.

For the ERP analysis, the amplitude and latency of P300 are examined. For the EEG data, a preprocessing process was conducted to eliminate the artifacts and interference in the data. During the experiment, 64 channels of EEG data were collected, among which CB1 and CB2 were unique channels of Neuroscan and were not included in the 10–20 system, so they were excluded. The reference channels M1 and M2 were also not included in the analysis. A 1–40 Hz bandpass filtering was performed on the 60-channel data to filter out power frequency interference and most noise. Then, the EEG data were divided into 130 segments based on the locations of the stimulation markers, of which 10 segments were in a resting state and the remaining 120 segments were at low, medium, and high stimulation levels. For those 120 segments of stimulation data, 1,000 ms and 4,000 ms of data were retained before and after the markers. A baseline correction was performed on the 120 segments of data based on the 1,000 ms data before the markers. At last, an Independent Component Analysis (ICA) was used to remove the artifacts from the EEG data, such as head movement, eye electrogram, and EMG.

All statistical analyses were performed using R software (Version 4.2.2, The R Foundation). For all analyses with *p* value smaller than 0.05 was considered statistically significant. A Shapiro–Wilk test was first used to check the sample normality. If the sample normality was not confirmed, a Friedman test was used to determine the significant difference among those data groups. If the Friedman test shown significant differences between the data sets, a Wilcoxon test was used to test for pairwise differences in the data sets. If the sample normality was confirmed, a Levene test was used to examine the homogeneity of variance. ANOVA was used to determine the significant difference among those groups. A two-tailed pairwise student t-test was used to compare the attention level difference between every two modes. When more than two groups of data were compared in this multiple hypothesis testing, a Benjamini-Hochberg method was used to control the false discovery rate.

## 3. Results

### 3.1. Recognition accuracy

**Figure 8:**
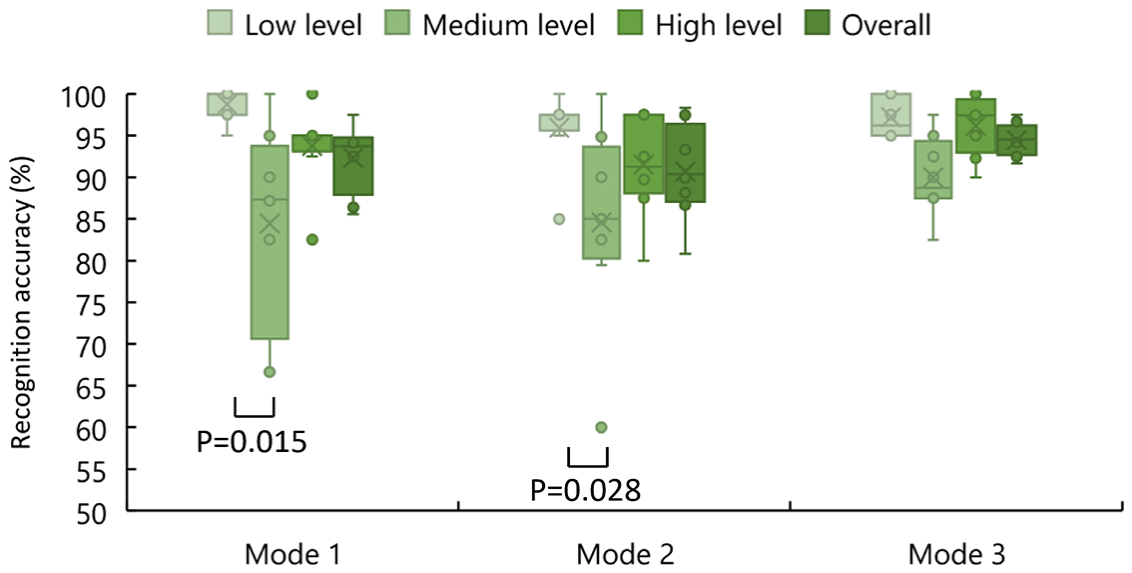
The recognition accuracies of stimulation levels.

Figure 8 shows the recognition accuracy of different stimulation levels. As one can see, the participants had the highest recognition accuracy for the low level of stimulation and the most insufficient recognition accuracy for the medium level of stimulation with a large individual difference. The sample normality was not confirmed for all the samples. Therefore, a Friedman test was used. For different modes, no significant difference was found (*p* = 0.658). In other words, the change of experimental modes had no significant effect on the recognition accuracy of stimulation levels. The different stimulation levels of Mode 1 had significant differences in recognition accuracy (*p* = 0.015). Specifically, low and medium stimulation levels in Mode 1 are significantly different in recognition accuracy (*p* = 0.005). Significant differences were also found in different stimulation levels of Mode 2 (*p* = 0.028) while no significant difference was found in different stimulation levels of Mode 3 (*p* = 0.093). Specifically, low and medium stimulation levels in Mode 2 are significantly different in recognition accuracy (*p* = 0.025).

### 3.2. Response time

**Figure 9:**
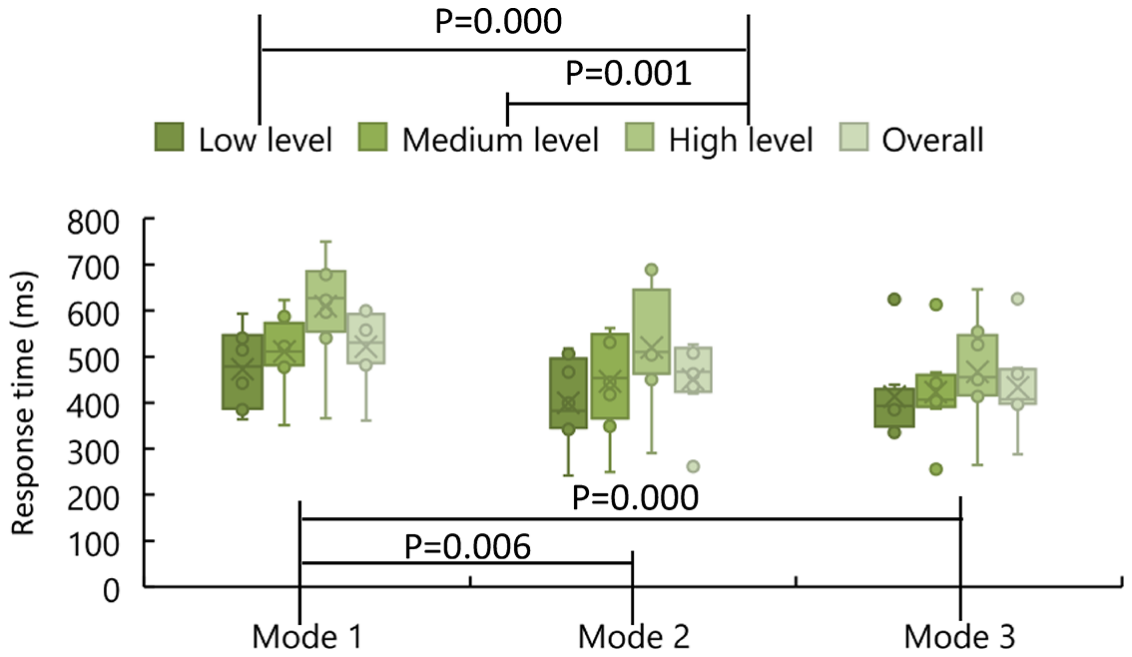
The response time of stimulation level recognition.

Figure 9 shows the response time for the participants to recognize the stimulation level. One can see that the average response time of the low level of stimulation was the shortest (Mode 1: 472.9 ms, Mode 2: 399.2 ms, Mode 3: 413.4 ms) while that of the high level of the simulation was the longest for all the three modes (Mode 1: 609.0 ms, Mode 2: 519.8 ms, Mode 3: 467.3 ms); the overall average response time of Mode 3 (433.1 ms) was the shortest while that of Mode 1 (521.8 ms) was the longest. The sample normality was confirmed for all the samples. The homogeneity of variance of all the samples was confirmed by a Levene test (*p* = 0.308). According to the results of the multi-factor ANOVA, the average response time of the participants was significantly affected by experimental modes (*p* = 0.001) and stimulation levels (*p* = 0.005), but there was no interaction between these two (*p* = 0.948). In the multiple pairwise comparisons, significant differences were found between low stimulation level and high stimulation level (*p* = 0.000), between medium stimulation level and high stimulation level (*p* = 0.001), but not between low stimulation level and medium stimulation level (*p* = 0.070). Significant differences were found between Mode 1 and Mode 2 (*p* = 0.018), between Mode 1 and Mode 3 (*p* = 0.000), but not between Mode 2 and Mode 3 (*p* = 0.441). In other words, regarding the response time, exoskeleton-assisted grasping motion with haptic stimulation was the same as grasping a water bottle but significantly different from that without haptic stimulation.

### 3.3. ERP analysis

**Figure 10:**
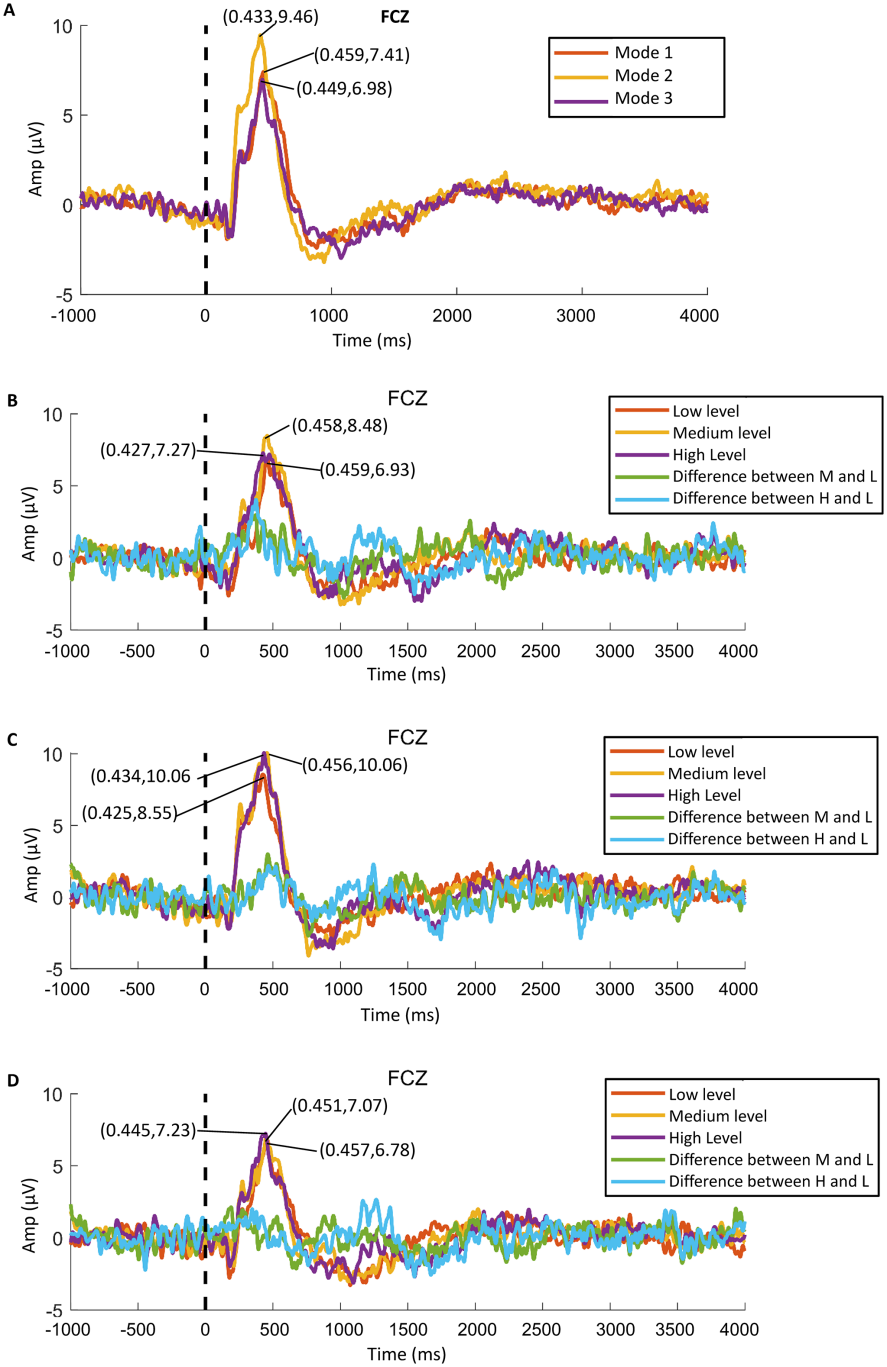
P300 amplitude data of FCZ channel: **(A)** average data of the three modes, **(B)** data of Mode 1, **(C)** data of Mode 2, and **(D)** data of Mode 3.

**Figure 11:**
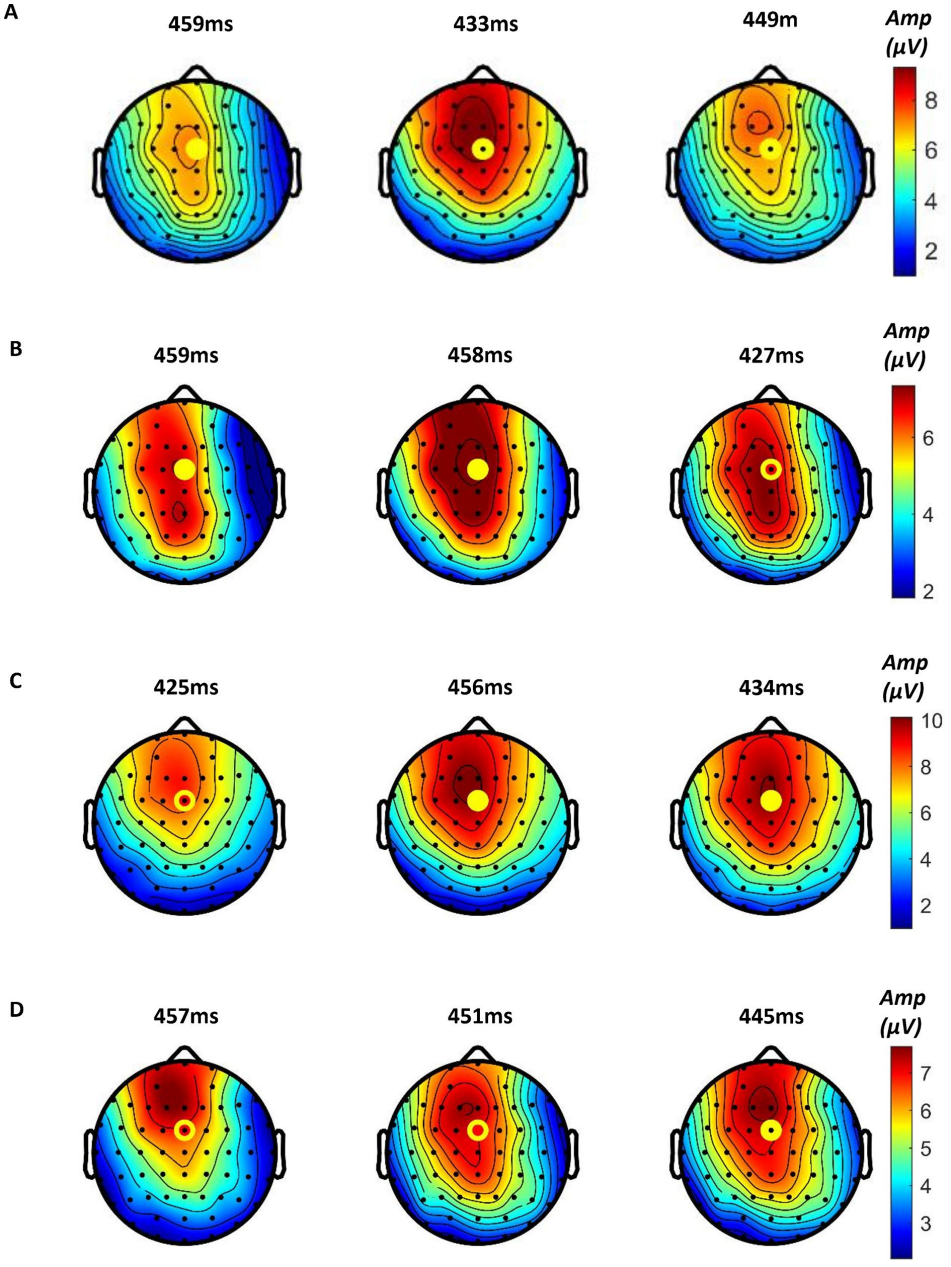
Topographical view of P300 amplitude at peak time of **(A)** the three modes (from left to right: Mode 1, Mode 2, and Mode 3), **(B)** Mode 1, **(C)** Mode 2, and **(D)** Mode 3 (from left to right: low stimulation level, medium stimulation level, and high stimulation level).

We found that all participants showed obvious ERPs throughout the brain in all samples. In order to further analyze the ERP signals, all the samples of each mode were processed by stacking average to extract the ERP components. Figure 10A shows the average P300 amplitude of the FCZ channel data of the three modes. One can see that the P300 amplitude of Mode 2 was the highest, followed by mode 1 and the lowest in Mode 3 (9.46 μV > 7.41 μV > 6.98 μV); the P300 latency was the shortest in Mode 2, followed by Mode 3 and the longest in Mode 1 (433 ms < 449 ms < 459 ms). Figure 11A shows the topographical view of P300 amplitude at peak of the three modes. It can be found that the CPZ-FZ region of the brain showed obvious activation in all three modes, the activation degree was the largest, and the activation area was the most extensive in Mode 2. In other words, the primary motor cortex (M1), premotor cortex (PM), and primary somatosensory area (S1) of the brain were more activated when both the hand motion assistance and fingertip haptic feedback are provided using our proposed method.

**Figure 12:**
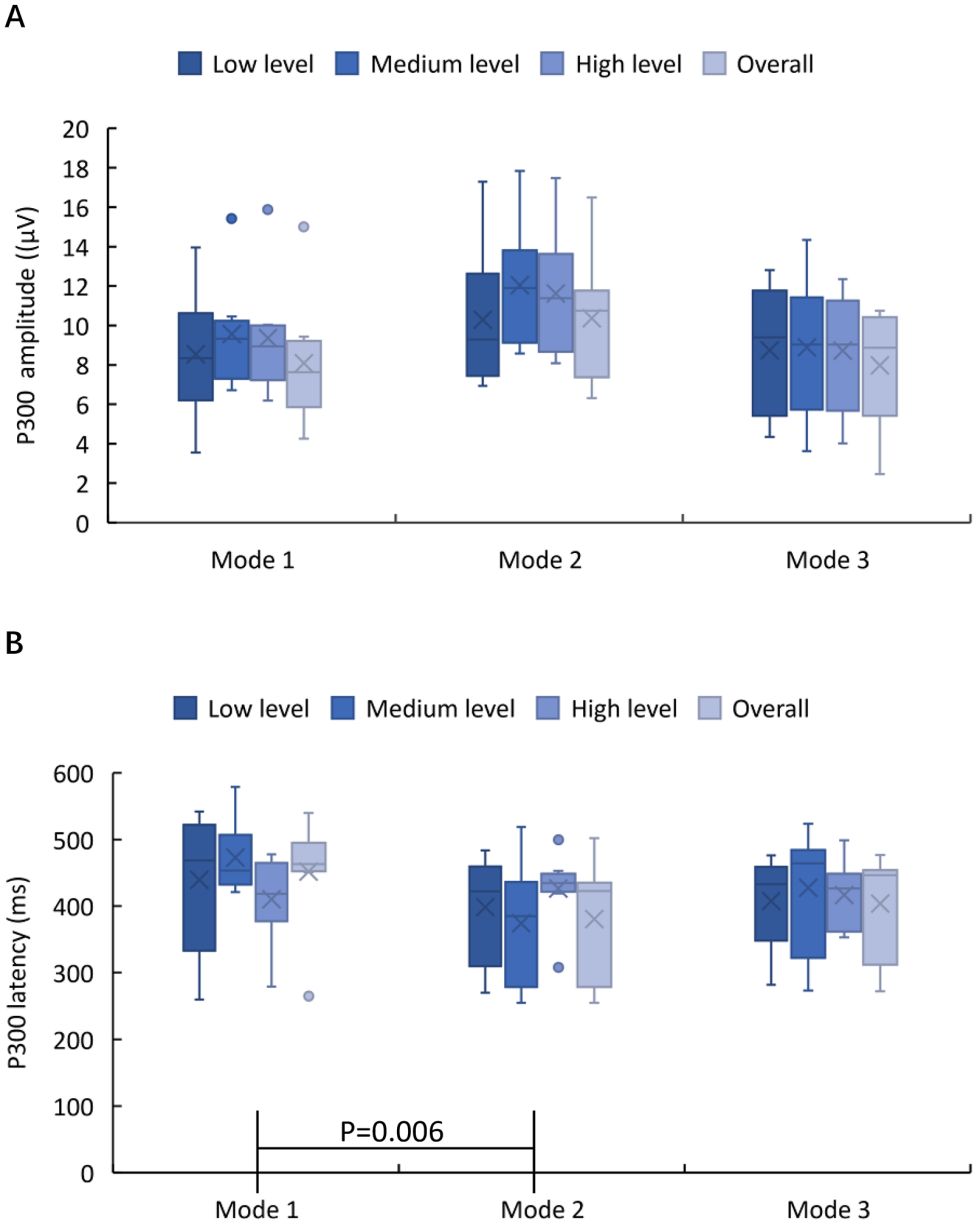
P300 amplitude, shown in **(A)**, and latency, shown in **(B)**, of the FCZ channel data of the three experimental modes.

Figure 12 shows the P300 amplitude and latency of the FCZ channel data. The sample normality was confirmed for P300 amplitude data (*p* = 0.223, *p* = 0.521, *p* = 0.133) but not for P300 latency data (*p* = 0.007, *p* = 0.161, *p* = 0.011). The homogeneity of variance of the P300 amplitude data was confirmed (*p* = 0.965). The results of the student t-test results showed that the amplitude of P300 in Mode 2 was significantly different from that in Mode 1 (*p* = 0.006), but no significant differences were found between any other two modes (Mode 2 vs. Mode 3: *p* = 0.227, Mode 1 vs. Mode 3: *p* = 0.918). In other words, the P300 amplitude was significantly improved by providing both exoskeleton-assisted hand motion and fingertip haptic stimulation compared to only providing exoskeleton-assisted hand motion; exoskeleton-assisted grasping motion with haptic stimulation evoked the same level of P300 amplitude as exoskeleton-assisted grasping motion with a water bottle. The Friedman test results showed that different modes did not significantly affect the P300 latency (*p* = 0.102).

Further, the in-group data of the three modes were analyzed to study whether different levels of stimulation using the same mode had different effects on relevant brain regions. Figures 10B–D show the P300 amplitude of the FCZ channel data in Mode 1, Mode 2, and Mode 3, respectively. Figures 11B–D show the topographical view of P300 amplitude at peak time in Mode 1, Mode 2, and Mode 3, respectively. For Mode 1, the P300 amplitude of the medium stimulation level was the highest, followed by the high stimulation level (8.48 μV > 7.27 μV > 6.93 μV); the P300 latency of the high stimulation level was the shortest, and that of the low stimulation level was the longest (427 ms < 458 ms < 459 ms). For Mode 2, the P300 amplitudes of the medium and high stimulation levels were the same (10.06 μV = 10.06 μV > 8.55 μV); the P300 latency of low stimulation level was the shortest, and that of medium stimulation level was the longest (425 ms < 434 ms < 456 ms). For Mode 3, the P300 amplitude of the high stimulation level was the highest, followed by the medium stimulation level (7.23 μV > 7.07 μV > 6.78 μV); the P300 latency of the high stimulation level was the shortest, and that of low stimulation level was the longest (445 ms < 451 ms < 457 ms). Table 2 shows the statistical analysis results of those data. No significant difference was found among the stimulation levels regarding P300 amplitude and latency. Therefore, the stimulation level had no effect on the P300 amplitude and latency.

**Table 2:** The statistical analysis results of P300 amplitude and latency of different levels of stimulation with the same stimulation mode.

Mode	Parameter	Sample normality	Homogeneity of variance	Test method	Result
Mode 1	P300 amplitude	Sample normality confirmed (*p* = 0.998, *p* = 0.067, *p* = 0.074)	Homogeneity of variance confirmed (*p* = 0.920)	One-way ANOVA (*p* = 0.295)	No significant difference
Mode 1	P300 latency	Sample normality not confirmed (*p* = 0.048, *p* = 0.153, *p* = 0.311)	Null	Friedman test (*p* = 0.417)	No significant difference
Mode 2	P300 amplitude	Sample normality confirmed (*p* = 0.206, *p* = 0.265, *p* = 0.612)	Homogeneity of variance confirmed (*p* = 0.944)	One-way ANOVA (*p* = 0.414)	No significant difference
Mode 2	P300 latency	Sample normality not confirmed (*p* = 0.037, *p* = 0.739, *p* = 0.033)	Null	Friedman test (*p* = 0.197)	No significant difference
Mode 3	P300 amplitude	Sample normality confirmed (*p* = 0.520, *p* = 0.959, *p* = 0.354)	Homogeneity of variance confirmed (*p* = 0.932)	One-way ANOVA (*p* = 0.867)	No significant difference
Mode 3	P300 latency	Sample normality not confirmed (*p* = 0.196, *p* = 0.018, *p* = 0.626)	Null	Friedman test (*p* = 0.607)	No significant difference

## 4. Discussion

The primary somatosensory cortex is closely related to the primary motor cortex, and the two are inseparable in function (Gómez et al., 2021). After a stroke, the motor-perception loop in patients is disrupted, and the synaptic connection between the perceptual and motor circuits in the brain is blocked. Due to the lack of sensory feedback, hand movement patterns continue to appear incorrect, leading to further worsening of hand sensory and motor dysfunction (Edwards et al., 2019). In clinical practice, cortical activity caused by somatosensory input is also used to predict late motor function recovery (Borich et al., 2015). Therefore, in the rehabilitation training process, not only should patients be assisted with motor rehabilitation, such as hand exoskeleton-assisted training, but also sensory stimulation should be applied simultaneously, including visual and tactile stimulation, to provide patients with timely and correct behavioral guidance and feedback (He et al., 2022). Through repeated correct sensory and motor stimulation, the motor-perception loop in patients can be naturally reshaped. By synchronizing the haptic stimulation with the motion of our hand exoskeleton, we expect to see the enhancement of the stimulation effect to both the motor and sensory cortex of the brain. In our previous study, we presented the creation and validation of a fingertip cutaneous haptic stimulation system for exoskeleton-assisted hand rehabilitation to improve the training involvement of stroke patients and promote motor function recovery (Li et al., 2021). The experimental results confirmed that adding haptic stimulation to exoskeleton-assisted hand movements significantly increased the attention levels of the participants. This paper presents a comparison study to further explore the stimulation enhancement effects of the combination of exoskeleton-assisted hand rehabilitation and fingertip haptic stimulation using both behavioral and ERP analysis. Moreover, the stimulation effects of the touch sensations created by a water bottle and that created by cutaneous fingertip stimulation with pneumatic actuators are compared to provide better guidelines for VR-mediated rehabilitation training. However, we have not linked the enhanced stimulation effects with evidenced improved hand function of stroke patients in the current study. In the future, clinical studies are required to further investigate this.

In the experiments, three experimental modes were compared, including exoskeleton-assisted grasping motion without haptic stimulation, exoskeleton-assisted grasping motion with haptic stimulation, and exoskeleton-assisted grasping motion with a water bottle. The experimental results showed that the change of experimental modes had no significant effect on the recognition accuracy of stimulation levels. Regarding the response time, exoskeleton-assisted grasping motion with haptic stimulation was the same as grasping a water bottle but significantly different from that without haptic stimulation. The ERP analysis showed that M1, PM, and S1 areas of the brain were more significantly activated when both the hand motion assistance and fingertip haptic stimulation were provided using our proposed method compared to hand motion assistance alone. Different modes did not significantly affect the P300 latency. The P300 amplitude was significantly improved by providing both exoskeleton-assisted hand motion and fingertip haptic stimulation compared to only providing exoskeleton-assisted hand motion; no significant differences were found between any other two modes. Thus, the above results confirmed the assumption that combining exoskeleton-assisted hand motion and fingertip haptic stimulation provided stronger stimulation on motor cortex and somatosensory cortex of the brain simultaneously. In addition, the above results also proved that the stimulation effects of the touch sensations created by a water bottle and that created by cutaneous fingertip stimulation with pneumatic actuators are similar. In the future, more studies are required to further investigate the mechanism and reasons.

By synchronizing the haptic stimulation with the motion of our hand exoskeleton, we expect to see the enhancement of the stimulation effect to the brain. In this study, this stimulation enhancement effect has been confirmed by the experimental results. Potentially, this haptic hand exoskeleton can be used in rehabilitation training that can provide stronger stimulation to the motor cortex and somatosensory cortex of the brain during treatment. In our previous study, we proved that adding haptic stimulation to exoskeleton-assisted hand movements significantly increase the attention levels of the participants. The increased attention levels of the participants may suggest an increase in the participants’ active involvement during the exoskeleton-assisted motion training process. Further, the increased active involvement of the participants combined with the enhancement of the stimulation effect on the brain may lead to better training outcomes. This should be investigated in future clinical studies.

Our previous study used a fingertip stimulation method to imitate the contact force of grasping a glass during an exoskeleton-assisted glass-grasping motion (Li et al., 2021). The fingertip haptic stimulation was added when the hand touched the simulated virtual glass. During this process, the change of the generated contact force was consistent with that when the hand holds a real glass. Since ERPs are time-sensitive, in this study, we altered the system design by activating both the hand exoskeleton and the fingertip haptic stimulation instantly in the experiment, which is different from what was used in our previous study (Li et al., 2021), to better evoke ERP components. The influence of this modification should be investigated in our future studies. Whether the conclusions drawn from our previous study can still hold true here also need further investigation. In this study, only P300 amplitude and latency, which were used for quantitative analysis of the somatosensory response to cutaneous haptic stimulations in literatures (Zhang et al., 2016; Chen and Ge, 2017; Tang et al., 2020), were examined. In the future, other EEG analysis methods that can quantitatively analyze the somatosensory response to haptic stimulations should be explored.

In our previous study, we found that haptic stimulation intensity significantly influenced on the evoked attention levels (Li et al., 2021). In this study, regarding the behavior analysis, both the recognition accuracy and the response time were significantly affected by haptic stimulation intensity. However, we found that stimulation intensity had no effect on the P300 amplitude and latency. The reason haptic stimulation intensity has significant effects on evoked attention levels, recognition accuracy, and response time but no significant influence on P300 amplitude and latency should be investigated in the future study. What is more, in the present experiment, only a group of young, healthy people participated. However, most stroke patients are with larger ages. Since human tactile perception declines with age (Chancel et al., 2018; Higgen et al., 2020), older patients may require greater haptic stimulation intensity to achieve the same evoked potential levels as younger subjects. In future studies, more stroke patients with different ages should be included to further prove the clinical feasibility of the proposed method.

## Data availability statement

The raw data supporting the conclusions of this article will be made available by the authors, without undue reservation.

## Ethics statement

The studies involving human participants were reviewed and approved by Institutional Review Board of Xi’an Jiaotong University (approval no. 2019-584). The patients/participants provided their written informed consent to participate in this study.

## Author contributions

ML: conceptualization, methodology, and supervision. ML, JC, and GH: hand exoskeleton design. ML and JC: experiment design. JC, BH, and GH: investigation. ML, JC and BH: data analysis. ML, JC, BH, GH, C-GZ, HY, JX, GX, and JL: writing. ML and GX: funding acquisition. All authors contributed to the article and approved the submitted version.

## Funding

This work was partially funded by the National Natural Science Foundation of China under grant (51975451) and the Key Research and Development Program of Shaanxi (program no. 2023-YBGY-353).

## Conflict of interest

The authors declare that the research was conducted in the absence of any commercial or financial relationships that could be construed as a potential conflict of interest.

## Publisher’s note

All claims expressed in this article are solely those of the authors and do not necessarily represent those of their affiliated organizations, or those of the publisher, the editors and the reviewers. Any product that may be evaluated in this article, or claim that may be made by its manufacturer, is not guaranteed or endorsed by the publisher.
